# The Future Availability of Family Caregivers: Implications for Late-Life Care Gaps

**DOI:** 10.1007/s11113-026-10016-4

**Published:** 2026-06-01

**Authors:** Vicki A. Freedman, Rachel Margolis, Ashton M. Verdery, Emily M. Agree, Esther M. Friedman

**Affiliations:** 1 University of Michigan–Ann Arbor, Ann Arbor, USA; 2 University of Western Ontario, London, ON Canada; 3 Pennsylvania State University, State College, USA; 4 Johns Hopkins University, Baltimore, USA

**Keywords:** Population aging, Caregiving, Family demography, Projections, Long-term care

## Abstract

Discussions about future care needs of U.S. older adults have frequently called attention to ongoing changes in families as the harbinger of a looming family care shortfall. To explore this premise, we paired estimates from the National Health and Aging Trends Study with projections of kinship patterns by age, sex, race through 2040. We defined care gaps in two complementary ways: the number of older adults with care needs who have (1) no family caregivers and (2) unmet care needs, whether they have caregivers or not. We projected outcomes for the overall older adult population and by sex, marital status, and racial groups, accounting first for only population growth and then allowing shifts in age structure, family structure, and family size. We projected that because of population growth alone, by 2040 the number of U.S. older adults with care needs but no family caregivers will reach 7.7 million and the number with unmet care needs will reach 14 million, up 22.9% from 2022 figures. Population growth and aging together resulted in a 32.5% and 33.9% increase, respectively, in these numbers. Impending changes in family structure and family size did not appreciably increase these estimates. Patterns for subgroups were substantially similar. We conclude that care gaps are projected to grow substantially in the United States by 2040, but contrary to prevailing concerns, population growth and changes in the age structure of the older population are primarily responsible, rather than shifts in family structure and family size.

**Supplementary Information:**

The online version contains supplementary material available at 10.1007/s11113-026-10016-4.

## Introduction

Although not an inevitable consequence of aging, the need for assistance with daily activities increases with age. Most family caregivers to older adults are spouses and children, but other family members and friends also play important roles, as do paid (non-family) caregivers (Freedman & Wolff, 2020). In 2022, more than 12 million older adults in the United States managed their basic health and functioning needs each day with the help of family (broadly defined) caregivers, an increase from 9 million a decade ago (Wolff et al., 2025). Over this same period, the number of family caregivers assisting older adults kept pace, increasing from 18 million to 24 million. Family caregivers assist older adults with a host of essential tasks including personal care (e.g. bathing, dressing), mobility (e.g. getting around inside and outside), household-related activities (e.g. preparing meals, doing laundry, running errands), transportation, and health-care activities (e.g., accompanying to health care appointments, managing medications).

The aging of the U.S. Baby Boom cohort, coupled with ongoing changes in demographic phenomena shaping families, have prompted concerns about a looming family care shortfall for older adults (National Academies of Sciences, Engineering, and Medicine, 2016). Researchers have pointed to the growing number of older adults who are embedded in stepfamilies (Patterson et al., 2022) and who are “kinless”—that is, with no spouse or children (Margolis & Verdery, 2017)—as particular concerns likely to inflate the future number of older adults with care needs but lacking family caregivers. Some demographic groups—for instance, women, Black, and unmarried older adults—have elevated risks of needing care in later life (Freedman, 2018), making future family structure for these groups especially important to understand.

Irrespective of whether an older adult has one or more family caregivers, in the United States about one-third of older adults with care needs experience adverse consequences related to inadequate help (Beach et al., 2017; Freedman & Spillman, 2014). Unmet care needs in later life are associated with greater risk of a variety of negative outcomes including emergency room visits, hospitalization, and death (Hass et al., 2017; He et al., 2015; Xu et al., 2012). Recent studies suggest lacking a spouse is related to a higher risk of unmet care needs, but associations with other aspects of family structure are less clear (Patterson et al., 2022; Patterson & Freedman, 2025). Risks of unmet care needs are higher for women than men, for unmarried older adults, and for older adults from minority backgrounds (Beach et al., 2017; Freedman, 2018).

In this paper we explore the influence of impending shifts in families of older adults on future late-life care gaps. We pair estimates of number and type of family caregivers and unmet care needs by age group, family structure and family size with projected kinship patterns through 2040. We project outcomes for the overall older adult population and key subgroups (men, women, married, unmarried, White and Black older adults), accounting first for only population growth and then allowing shifts in age structure, family structure, and family size.

## Background

### Changes in the Structure of Families of Older Adults

Recent and future trends in the families of U.S. older adults result from decades-long shifts in fertility and partnering/re-partnering patterns. The rise of two demographic phenomena among older adults—kinlessness and stepfamilies—are of particular interest because both trends are likely to contribute to fewer family caregivers available to help older adults with care needs.

Although classification approaches vary, kinlessness has often been defined as simultaneously lacking a spouse and biological children (Margolis & Verdery, 2017). The percentage of kinless U.S. older adults has been increasing as younger cohorts reach late life (Margolis & Verdery, 2017). For instance, in 2015, about 7−8% of White and 10−11% of Black adults ages 55 or older were kinless; these figures are expected to reach 8−10% for White and 12−13% for Black older adults by 2040 (Verdery & Margolis, 2019). These impending increases are notable because spouses and adult children are most likely to provide care to older family members with care needs (Wolff et al., 2025).

A second trend of interest is the coming increase in the number of older adults living in stepfamilies (Lin et al., 2018; Patterson et al., 2022). Estimates of older adults embedded in stepfamilies have varied depending on the unit of interest. One study found that in 2012, 40% of couples in which at least one partner was age 51 or older were in stepfamilies (Lin et al., 2018); another study focused on parents ages 65 or older in need of care found that in 2015 14% had at least one stepchild (Patterson et al., 2022). Irrespective of current estimates, researchers expect the percentage of older adults living in step-families to “surge” in the coming decades due to high rates of divorce and re-partnering in midlife and older ages and more favorable attitudes toward post-divorce cohabitation, since most individuals in these unions have children from prior relationships (Lin et al., 2018). Moreover, among older couples, older adults from minority groups have made up a disproportionate share of later-life stepfamilies (Lin et al., 2018). Relative to those in biological families, older adults with care needs who have stepfamily members have been less likely to receive assistance from a family member, particularly when there were no joint biological children (Patterson et al., 2022; Schoeni et al., 2022) and their care was divided less equally among siblings (Lin & Wolf, 2022).

### Studies Suggesting an Impending Care Crisis

Often cited as evidence of an impending shortage of family members to provide late-life care is the steep anticipated decline in a measure referred to as the caregiver support ratio (Redfoot et al., 2013). This ratio compares the number of people in the U.S. population ages 45−64, a proxy for potential caregivers, to the number ages 80 and older, a proxy for potential care recipients. This ratio is expected to decline by half from about 6 in 2020 to 3 in 2040 (Redfoot et al., 2013).

Although straightforward to calculate, the caregiver support ratio does not provide an accurate picture of the future availability of family care. For instance, the numerator leaves out a substantial fraction of caregivers who are ages 65 or older or younger than age 45 (roughly one-half based on Wolff et al., 2025), and the denominator includes a substantial fraction of adults ages 80 and older who do not necessarily need care (roughly one-half based on Freedman, 2018). Additionally, because caregivers and recipients may come from different demographic groups, the caregiver support ratio is not optimal for exploring differences between men and women or for groups with elevated risks for care gaps (Patterson & Freedman, 2025; Beach et al., 2017).

Microsimulation models that build kinship networks over time starting from an initial population are a helpful tool to characterize future kin availability of the older population as a whole and among key subgroups. Recently, Wu et al. (2024) paired microsimulation model results with data from the Health and Retirement Study to explore the implications of demographic and family changes for care gaps, which they defined as receiving no help (from either family, other unpaid or paid sources) with a reported difficulty in the last three months. Applying rates from 1998 to 2014 to future projected population distributions by race, sex, age, and family structure, they found care gaps among adults ages 50 and older will increase by more than 30% between 2015 and 2050—twice the rate of population growth. They also found that gaps were higher among those with no spouse or biological children, suggesting that impending family changes may be in part driving these increases.

Importantly, the article left open several questions relevant to meeting the future care needs of the aging population. First, the authors projected only the most common family structure categories, reflecting combinations of availability of a spouse and biological children, and assessed only simultaneous changes in family structure and demographic factors. Consequently, it remains unclear how much of the projected changes are the result of shifts in family composition and family size. Second, the analysis was not able to provide insights into implications of changing families for the type (e.g., paid vs. family caregivers) or number of caregivers assisting older adults in the future. Third, it was unclear whether using alternative measures of care gaps would yield similar conclusions. As the authors noted, conceptually, unassisted difficulty is both overly broad—because it includes large numbers of adults who address difficulties without help but instead with assistive devices, environmental modifications, or behavioral changes—and overly narrow— because it does not capture those receiving insufficient help who despite assistance have unmet care needs (Freedman, 2018). Empirically, measures of unassisted difficulty and unmet care needs have identified groups that differ in both size and composition (Patterson & Freedman, 2025). For instance, among those reporting difficulty with self-care or mobility activities in the last month, 80% did not receive help whereas 29% experienced an adverse consequence related to unmet care needs (Patterson & Freedman, 2025).

### The Current Study

In this paper, we have built on this literature by pairing probabilities from the National Health and Aging Trends Study (NHATS) with projected population counts capturing future kinship patterns that include both biological children and stepchildren. We have explored the influence of shifting age structure, family structure, and family size on the number of adults ages 65 and older experiencing care gaps. We have used two measures of care gaps among older adults with care needs: (1) having no family caregivers; and (2) having an unmet need for care. Altering one factor at a time, we have focused on changes in these care gap measures from 2022−2040, a period of steep decline in the caregiver support ratio. Our approach has allowed us to isolate the future influence of the changing demography of families on complementary measures of family care gaps.

## Data and Methods

This study drew upon two sources of data: NHATS for estimating probabilities of receiving care and having unmet need by family structure and size and a demographic microsimulation model to project family structure and size.

NHATS drew its sample from the Medicare enrollment file, with oversamples of beneficiaries at older ages and Black individuals (Freedman & Kasper, 2019). The study was initiated in 2011 and the sample was replenished in 2015 and in 2022. Response rates were 71% in 2011, 77% in 2015, 59% in 2022, and for intervening years ranged from 86% to 97% (Freedman et al., 2024). Weights that account for differential selection and nonresponse probabilities were provided to researchers so that estimates of the 65 and older Medicare population (approximately 96% of all older adults in the United States) could be generated.

Altogether 5,900 individuals were eligible and completed a detailed interview in 2022. Because older adults living in nursing homes in the year that they entered NHATS were not eligible for an interview, we omitted individuals who moved into a nursing home setting after entering NHATS $\:(n$ = 66). To align with simulation assumptions, we omitted 20 cases older than age 100, 817 individuals who reported being born outside the United States, and 450 U.S.-born individuals who reported a race/ethnicity other than White non-Hispanic or Black non-Hispanic (hereafter, White and Black), yielding a sample of $\:N$ = 4,547 individuals for main analyses. We also created a sample that included older adults who were foreign born and other race/ethnicities ($\:N$ = 5,814) to explore alternative projections with the full sample. From the main sample we also created subsamples for men ($\:n$ = 1,903) and women ($\:n$ = 2,644), for married ($\:n$ = 2,083) and unmarried ($\:n$ = 2,464), and for White ($\:n$ = 3,459) and Black ($\:n$ = 1,088) older adults.

The demographic microsimulation used in this analysis simulated historical and projected kinship networks until 2040 (Verdery & Margolis, 2017). For the historical period, it drew upon separate starting populations for White and Black (non-Hispanic) individuals, matching population distributions in these groups in the 1880 U.S. Census. These populations and their simulated descendants then experienced fertility, mortality, and family building events consistent with the historical record through 2017 (up to age 100). To simulate changes to 2030, the microsimulation used the demographic assumptions of the U.S. Census Bureau for its most recent National Projections, which offer the only recent source of age-, race-, ethnicity-, and nativity-specific demographic rates for 2030 and beyond (U.S. Census Bureau, 2017). The rates used include: race-age-sex-specific mortality, marriage and remarriage rates; race-duration-specific divorce rates; and (for women) race-age-parity-marital status-specific fertility rates. Simulating individuals in this fashion produced life history and kinship profiles, which we summarized at the population level. Prior work has found these profiles align well with empirical data (Verdery et al., 2020). In this application, the resulting age-sex-race groups were scaled to match totals for the 65 and older U.S.-born population in 2022, 2030, and 2040 (Verdery & Margolis, 2017). Using these simulated populations, we then characterized family structure and family size (described below) in each year.

### Measures

*Care and Unmet Need* NHATS participants were asked how they performed self-care and mobility tasks (eating, bathing, toileting, dressing, getting around inside, and getting out of bed) and household activities (doing laundry, preparing meals, shopping, paying bills, and handling medications) in the last month. All helpers were recorded along with information about their relationship to the sample person and whether they were paid. The reason help was received (e.g. health or functioning, other reason) was also recorded for household activities. From this information we created an indicator reflecting the number (0 through 3 or more) of relatives and other unpaid caregivers (hereafter, “family caregivers”) assisting for health or functioning reasons and an additional category indicating reliance on only paid non-family caregivers.

NHATS also asked about adverse consequences associated with unmet care needs. Respondents were asked whether in the last month they had experienced a particular consequence related to the absence of help (e.g., having to stay in bed; going without showering, bathing, or washing up). From this information we created a summary measure indicating any unmet need.

For both outcome measures we further distinguished individuals who had no care needs. Individuals who either reported receipt of help with self-care, mobility or household activities (the latter for health or functioning reasons) or an unmet need for help with any of these activities were considered to have care needs. Those who reported no difficulty or help with activities ($\:n$ = 2,023) or who reported difficulty but no indication there was an unmet need for help ($\:n$ = 891) were classified as not having care needs; we also explored sensitivity of findings to a broader definition of care needs, where we reclassified the those with unassisted difficulty as having a care need even if there was no indication of an unmet need.

*Family Structure and Size* We created indicators of both family availability and family size in 2022. Family availability included six, mutually exclusive groups. Three groups were unmarried: one with no children, a second with only biological children but no stepchildren, and a third with any stepchildren (with or without biological children). Three additional groups were married (with no children, with only biological children, and with any stepchildren). Family size was constructed to reflect a count of biological children and stepchildren plus one if the older adult was married (range 0 to ≥ 4).

### Analytic Approach

We compared the 2022 NHATS population estimates to the simulated distributions for that year by age group, male/female, racial group and family structure and size. Age, sex, and racial groups aligned well between the two sources, but the fraction of older adults with stepchildren and with 4 or more family members was higher in the simulated data. We believe this finding likely signals under-reporting of stepchildren in NHATS, particularly from former marriages. We therefore realigned the weighted NHATS distributions to more closely match the 2022 projections (see Table S1 in the Online Supplement). For these calculations we assumed that the subgroup of respondents who changed categories (e.g., gained a stepchild, or moved into a larger family size) retained care and unmet need distributions of their original group, since we had no empirical basis for assuming systematic differences. To the extent that there was selection that we have not captured, our recalibrated proportions may be biased for some groups. All probabilities generated from NHATS were weighted (see Table S2 in the Online Supplement).

We multiplied the number of older adults in each subgroup from the population simulations in 2022, 2030, and 2040 with the corresponding 2022 subgroup probabilities from NHATS (i.e., with unadjusted probabilities stratified by either age group, family structure, or family size) and summed over each set of subgroups to obtain the expected number and percentage for each care and unmet need category. In these scenarios, changes in the numbers of older adults represented the contribution of population growth and the shift in each demographic factor of interest. This approach allowed us to illustrate the effect of shifts in family structure and family size on future distributions of care and unmet need and to compare these changes to those that would occur solely due to shifts in population growth and aging.

We also explored the effect of excluding individuals who were foreign born and from Hispanic and other racial and ethnic groups. Based on our analysis of NHATS, we assumed this group constituted 20% of the population in 2022 and that subgroups by age, family structure, and size grew in proportion to subgroups in the rest of the population through 2040 (see Table S3 in the Online Supplement). To further illustrate the influence of projected age structure, family structure, and family size, we calculated the distribution of the 65 and older population with care needs across caregiver categories (percentage with none, only paid, 1, 2, and 3 or more family caregivers) using the expanded sample and also calculated the mean number of caregivers among those with care needs. Finally, we made projections separately for men and women, married and unmarried, and White and Black older adults using the main sample. Subgroup-specific probabilities and projected characteristics are provided in the Supplementary material (see Tables S4 and S5 in the Online Supplement).

This analysis relied upon publicly available data and therefore did not require Institutional Review Board review.

## Results

The population of U.S. born White and Black adults ages 65 and older was projected to grow from 50.5 million in 2022 to 62.1 million in 2040 (see Table 1, Panel A). The aging of the older population was also evident, as the groups ages 75 to 84 and 85 and older increased by about 8 percentage points, whereas the youngest age group (ages 65 to 74) decreased from 57.0% to 41.7% as a percentage of the older population (see Table 1, Panel B). Although the sex composition was projected to be stable over this time period (45% men and 55% women), small increases in the percentage of Black older adults (11.3% in 2022 to 14.4% in 2030) were projected.

**Table 1:** Projected number and percentage of the U.S. Population ages 65 and older, 2022–2040, and percentage in 2022 with care gaps, by age, sex, race and family characteristics, 2022–2030

	A Number (Millions)			B Percentage			C Percentage in 2022 with care needs and:			
							No family caregiver			Unmet need for care
	2022	2030	2040	2022	2030	2040	All	No caregivers	Only paid caregivers	
Overall	50.5	59.9	62.1	100.0	100.0	100.0	11.0	9.7	1.3	15.1
*Age*										
65–74	28.8	30.9	25.9	57.0	51.6	41.7	9.4	8.3	1.1	13.2
75–84	15.7	21.2	24.2	31.1	35.5	39.0	12.9	12.1	0.8	16.0
≥ 85	6.0	7.7	12.0	11.9	12.9	19.3	13.3	9.3	4.0	22.7
*Sex*										
Men	22.7	27.0	27.9	45.0	45.2	45.0	11.1	9.8	1.3	12.8
Women	27.8	32.8	34.2	55.0	54.8	55.0	10.9	9.6	1.3	17.0
*Race*										
White	44.8	52.3	53.1	88.7	87.4	85.6	10.9	9.7	1.2	14.7
Black	5.7	7.5	9.0	11.3	12.6	14.4	11.6	9.6	2.0	18.7
*Family structure*										
*No spouse*										
…no biological children or stepchildren	2.5	3.4	3.9	5.0	5.8	6.2	17.3	13.3	4.0	20.2
…has biological children only	10.4	11.5	12.1	20.7	19.3	19.4	12.8	10.5	2.3	21.5
…has stepchildren	8.2	9.5	10.3	16.3	15.9	16.6	14.5	10.5	4.0	19.2
*Has spouse*										
…no biological children or stepchildren	2.6	3.7	3.5	5.1	6.2	5.6	10.9	10.9	0.0	8.8
…has biological children only	14.8	17.4	17.8	29.3	29.1	28.6	7.6	7.4	0.2	10.2
…has stepchildren	12.0	14.2	14.6	23.7	23.8	23.5	10.3	10.2	0.1	13.3
*Family size* ^a^										
0	2.5	3.4	3.9	5.0	5.8	6.2	17.3	13.3	4.0	20.2
1	5.1	7.0	7.2	10.0	11.7	11.7	10.1	9.7	0.4	15.4
2	7.8	9.4	9.8	15.4	15.8	15.8	13.0	11.0	2.0	17.2
3	10.0	11.6	11.8	19.7	19.4	19.0	8.4	7.6	0.8	12.7
≥ 4	25.2	28.4	29.4	49.8	47.4	47.3	10.9	9.7	1.2	14.9

Projections exclude foreign born and Hispanic older adults and other racial groups. ^a^Count of spouse, biological children, and stepchildren

The distribution of family structure of older adults in the United States was projected to shift slightly towards groups with fewer kin. The share of older adults without children was projected to increase (from 5.0% to 6.2% for unmarried and from 5.1% to 5.6% for married) as was the percentage of unmarried older adults with any stepchildren (16.3% in 2022 and 16.6% in 2040). Unmarried and married older adults with only biological children were projected to decline modestly (from 20.7% to 19.4% and from 29.3% to 28.6%, respectively) and the proportion with stepchildren was projected to remain stable at 40%. Larger, more common family sizes were projected to decline from 19.7% to 19.0% (for 3 family members) and from 49.8% to 47.3% (for 4 or more family members), whereas less prevalent family sizes of 0, 1 and 2 were projected to increase.

Overall, 11.0% of older adults in 2022 had care needs and no family caregiver (9.7% no caregiver of any kind and 1.3% only paid caregivers), but these percentages varied by both family structure and family size (see Table 1, Panel C). For instance, unmarried childless older adults were most likely to have no family caregiver (17.3%) whereas the figure for married older adults with only biological children was still sizable but nearly 10 percentage points lower (7.6%). Older adults with stepchildren were about 2−3 percentage points more likely than those with only biological children to have no family caregiver. Family size had a non-linear association with these outcomes; older adults with a family size of 0 were most likely to have no family caregiver (17.3%) whereas those with 3 family members were least likely (8.4%). Similarly, estimates of unmet need were 15.1% overall, but were highest for those with no spouse (19.2−20.2%, depending on presence of children) and with a family size of 0 (20.2%). Unmet need was about 2−3 percentage points higher for older adults with stepchildren relative to those with only biological children.

Table 2 presents two sets of projections: main projections that excluded foreign born older adults and racial ethnic groups other than White and Black and alternative projections that included these groups assuming they grow at the same rate as the rest of the population. For each projection set, we first calculated the number of older adults in the population in each year and the percentage increase from 2022 to 2040. We then calculated the number of older adults with limitations and no family caregiver due to (1) population growth only, and (2) population growth and changes in (a) age structure, (b) family structure, and (c) family size, respectively, followed by the number of older adults with limitations and unmet need due to these same factors. If changes in age structure, family structure, or family size contributed to care gaps, we would expect the percentage increase to be higher than the percentage increase for population growth only.

**Table 2:** Projected number of U.S. Adults ages 65 and older with care needs and no family caregiver and with unmet need due to population growth and changes in age, family structure and family size: 2022, 2030 and 2040 (in Millions)

	A Main projections					B Alternative projections			
	2022	2030	2040	% Increase 2022–2040		2022	2030	2040	% Increase 2022–2040
Population growth	50.5	59.9	62.1	22.9%		62.8	74.4	77.2	22.9%
*Changes in No family ca* *regiver due to:*									
Population growth only	5.5	6.6	6.8	22.9%		6.3	7.4	7.7	22.9%
& Age structure	5.5	6.7	7.1	29.3%		6.3	7.7	8.4	32.5%
& Family structure	5.5	6.6	6.9	23.9%		6.3	7.5	7.8	24.3%
& Family size	5.5	6.6	6.9	23.9%		6.3	7.5	7.8	24.5%
*Changes in Unmet need due to:*									
Population growth only	7.6	9.1	9.4	22.9%		11.7	13.9	14.4	22.9%
& Age structure	7.7	9.2	10.0	30.4%		11.9	14.4	15.9	33.9%
& Family structure	7.6	9.0	9.4	22.9%		11.9	14.1	14.6	23.4%
& Family size	7.6	9.1	9.5	23.7%		11.7	14.0	14.5	24.1%

Main projections exclude foreign born and Hispanic older adults and racial groups other than White, Black. Alternative projections include these groups and assume they grow at the same rate as the baseline population

Holding all factors constant except growth in the older population, the number of older adults with care needs but lacking family caregivers was projected to grow from 5.6 million in 2022 to 6.8 million in 2040, a 22.9% increase over nearly twenty years (see Table 2, Panel A). Allowing population growth and age structure to change resulted in a 29.3% increase—roughly one-fourth to one-third higher than population growth alone. Allowing population growth and either shifts in family structure or family size yielded figures in 2040 on par with population growth alone (6.9 million).

In alternate projections that incorporated foreign born individuals and those from racial and ethnic groups other than White and Black groups, we found substantially similar patterns to the main projections (see Table 2, Panel B). The number of older adults with care needs but lacking family caregivers was projected to grow from 6.3 million in 2022 to 7.7 million in 2040 (increasing by 22.9%). Population growth and aging together resulted in a 32.5% increase in the number of older adults with no family caregivers, respectively, nearly one-third higher than population growth alone. In contrast, population growth and family structure (size) yielded estimates in 2040 that were just slightly higher than population growth alone.

**Fig. 1:**
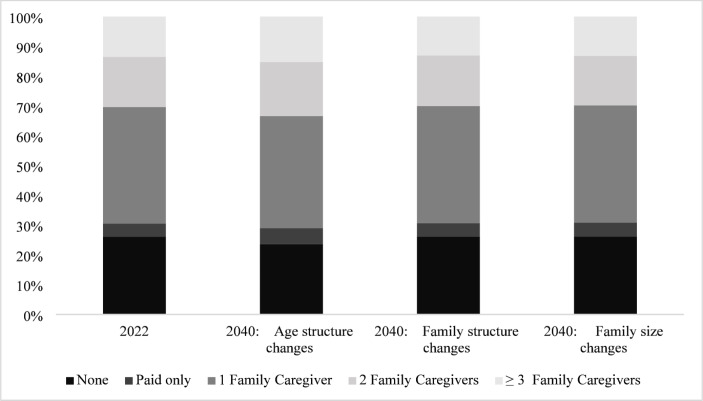
Percentage distribution of number of family caregivers among U.S. adults ages 65 and older with care needs, 2022 & 2040.* Note*: Mean number of family caregivers among those with limitations is 1.1 for 2022 and 2040 projections with family structure and family size changes and 1.2 for 2040 with age structure changes. See Table S7 in the Online Supplement for underlying estimates.

Similar patterns were evident for each category reflecting type and number of caregivers (no caregivers, only paid caregivers, 1, 2, and 3 or more family caregivers) for both main and alternative projections (see Table S6 in the Online Supplement). Re-expressing these alternative population projections as distributions across categories, we found in 2022 that more than 30% of older adults with care needs had no family caregivers (26.1% no caregivers plus 4.5% with only paid caregivers), 39.1%, 16.9%, and 13.5% had 1, 2, and 3 or more family caregivers, respectively, and the mean number of caregivers was 1.1 (see Fig. 1 and underlying estimates in Table S7 in the Online Supplement). Projections in 2040 that allowed age structure to vary yielded slightly higher percentages with 2 and 3 or more family caregivers, a higher percentage with only paid caregivers, and slightly lower percentages with no family caregivers and 1 family caregiver. Changes in family structure and size in 2040 yielded even smaller differences from 2022 than did changes in age structure, and in some cases, results were in the opposite direction. For instance, projections based on 2040 family structure and family size resulted in a lower percentage with 3 or more family caregivers than in 2022 (13.1% for both family structure and family size vs. 13.5% in 2022) and a higher percentage with 1 family caregiver (39.3% for family structure and 39.4% for family size vs. 39.1% in 2022). Family structure and size changes did not appreciably affect the projected percentage with no caregivers or with only paid care. The mean number of family caregivers per older adult with a limitation was projected to be marginally higher in 2040 as the result of age structure changes (mean = 1.2), but was unchanged from 2022 as the result of shifts in family structure and family size (mean = 1.1).

In sensitivity analyses, we used a broader definition of care needs that included all individuals reporting unassisted difficulty, whether they reported unmet care needs or not (see Table S8 in the Online Supplement). Results suggested a much larger number of older adults with care needs but without caregivers (e.g., nearly 18 million in 2040 rather than nearly 7 million), but conclusions about the minimal influence of changes in family structure and size in projections were robust. Projections of other estimates were not affected by the reclassification.

Focusing on projections of unmet need (see Table 2, Panel A), patterns with respect to population growth and aging were similar to those observed for type and number of caregivers. The number of older adults with unmet need was projected to grow by 22.9% from 7.6 million in 2022 to 9.4 million in 2040 as a result of growth of the older population. Population growth and changes in age structure together were projected to result in 10.0 million older adults with unmet need in 2040; a 30.4% increase, or between one-fourth and one-third higher than population growth alone. However, allowing population growth and shifts in family structure and size yielded projected numbers in 2040 on par with population growth alone (9.4 and 9.5 million, respectively). Alternative projections with the more complete population (see Table 2, Panel B) resulted in substantially similar findings to the main projections: the number of older adults with unmet need was projected to increase from 11.7 million to 14.4 million (22.9% increase), population growth and aging together resulted in a 33.9% increase, whereas changing population growth and family structure (size) yielded estimates in 2040 that exceeded those from population growth alone by about 100,000–200,000 (23.4% and 24.1% increases, respectively).

We replicated the main projections for older adults with care needs and no family caregiver by sex, marital status, and race (see Table 3, Panel A). Several findings are noteworthy. First, findings for men mirrored patterns for the overall population; that is, age structure shifts between 2022 and 2040 led to a 34.8% increase in the number of men with no family caregivers (from 2.5 to 3.3 million), but changes in family structure and size did not result in excess projected numbers beyond population growth alone (3.1 million; roughly 23% increase). Second, the group of 3.0 million women with no family caregivers was projected to grow to 3.7 million in 2040 due to population growth alone; incorporating changes in age structure, family structure and family size into projections led to a relatively small excess of about 100,000 individuals (from 3.0 to 3.8 million) beyond population growth for each factor separately. In terms of percentage increases, population growth alone resulted in a 22.8% increase whereas population growth and shifts in each of the factors led to 24.3%–25.3% increases. Third, projections for unmarried and Black older adults differed from married and White older adults with respect to the influence of population aging but not family structure or family size. Population aging had a substantial effect on the number of married and White older adults with no family caregiver (but not on the number of unmarried or Black older adults with no family caregiver); however, for all four groups, family structure and size did not result in excess numbers beyond population growth.

**Table 3:** Projected number of U.S. adults ages 65 and older with care needs and no family caregiver and with unmet need due to population growth only and changes in age, family structure and family size: men, women, married, unmarried, white and black adults in 2022, 2030 and 2040 (Millions)

	Projected number with care needs and:							
	A No family caregiver				B Unmet need			
	2022	2030	2040	% Increase	2022	2030	2040	% Increase
*Men*								
Population growth only	2.5	3.0	3.1	23.0%	2.9	3.5	3.6	23.0%
& Age structure changes	2.5	3.0	3.3	34.8%	2.9	3.5	3.7	27.7%
& Family structure changes	2.5	3.0	3.1	23.3%	2.9	3.5	3.6	23.1%
& Family size changes	2.5	3.0	3.1	22.9%	2.9	3.5	3.6	23.5%
*Women*								
Population growth only	3.0	3.6	3.7	22.8%	4.7	5.6	5.8	22.8%
& Age structure changes	3.0	3.6	3.8	25.3%	4.8	5.7	6.2	31.4%
& Family structure changes	3.0	3.6	3.8	24.3%	4.7	5.5	5.8	22.8%
& Family size changes	3.0	3.6	3.8	25.0%	4.7	5.6	5.8	23.7%
*Married*								
Population growth only	2.6	3.1	3.2	22.2%	3.2	3.9	3.9	22.2%
& Age structure changes	2.6	3.3	3.5	34.9%	3.1	3.9	4.0	28.5%
& Family structure changes	2.6	3.1	3.2	22.6%	3.2	3.8	3.9	22.1%
& Family size changes	2.6	3.1	3.2	22.6%	3.2	3.9	3.9	22.5%
*Unmarried*								
Population growth only	2.9	3.3	3.6	23.8%	4.3	5.0	5.3	23.8%
& Age structure changes	2.9	3.3	3.6	23.8%	4.4	5.1	5.7	28.9%
& Family structure changes	2.9	3.3	3.6	25.1%	4.3	5.0	5.3	23.6%
& Family size changes	2.9	3.3	3.6	24.2%	4.3	5.0	5.3	23.5%
*White*								
Population growth only	4.9	5.7	5.8	18.5%	6.6	7.7	7.8	18.5%
& Age structure changes	4.9	5.8	6.1	25.6%	6.6	7.8	8.3	26.5%
& Family structure changes	4.9	5.7	5.8	19.2%	6.6	7.6	7.8	18.3%
& Family size changes	4.9	5.7	5.9	19.6%	6.6	7.7	7.9	19.4%
*Black*								
Population growth only	0.7	0.9	1.0	57.8%	1.1	1.5	1.8	57.8%
& Age structure changes	0.7	0.9	1.0	57.5%	1.1	1.5	1.8	63.9%
& Family structure changes	0.7	0.9	1.0	58.2%	1.1	1.5	1.7	56.0%
& Family size changes	0.7	0.9	1.0	57.7%	1.1	1.5	1.7	57.3%

Projections exclude foreign born and Hispanic older adults and other racial groups

Projection patterns for unmet need across the subgroups (see Table 3, Panel B) were similar to those for having care needs and no family caregiver. Population growth alone was projected to lead to increases of 19%–23% for all groups except Black older adults, who were projected to have a 58% increase (from 1.1 million to 1.8 million) in the number with unmet needs. Age structure shifts led to larger increases, on the order of 5–9 percentage points higher. In contrast, family structure and family size did not appreciably change the number or percentage increase beyond population growth.

## Discussion and Conclusion

By 2040, because of population growth alone, 7.7 million adults ages 65 and older in the United States will have care needs but no family caregiver and more than 14 million will have an unmet need for care. The growth and aging of the population will exacerbate these changes, pushing up these numbers to 8.4 and nearly 16 million, respectively. Yet, contrary to prevailing narratives, ongoing changes in family structure or family size will not appreciably exacerbate the number with care gaps beyond what is anticipated as a consequence of population growth alone.

Despite different studies, age groups, time periods and outcomes of interest, our findings are largely consistent with those published by Wu et al. (2024). In that study, the authors found unassisted difficulty among adults ages 50 and older was projected to increase by more than 30% between 2015 and 2050 due to population growth and shifts in age structure, race, sex and kinlessness—twice the rate of population growth alone. Using two complementary measures that focus more narrowly on those needing help, we found that care gaps among those ages 65 and older were projected to increase by more than 30% between 2022 and 2040 due to population growth and shifts in age structure—nearly one-third higher than they would be based on population growth alone. However, by having created projections that alter only one factor at a time, we are able to conclude that impending shifts in family structure and size do not appreciably increase these projected gaps beyond what is expected related to population growth.

Our analysis also provides new insights into *why* the changing demography of families with respect to family size and composition is likely to have such a small influence on the size of the care network and unmet need. The percentage of unmarried, childless older adults is small in 2022 and projected to increase only marginally (from 5.0% to 6.2%), and the chances of having needs but no caregivers (17%) or unmet need (20%) is not substantially out of range from estimates for older adults with other family structures and sizes (8−15% and 9−22%, respectively). Similarly, projected declines in family size are driven not only by small increases in the percentage of older adults with no family members, but also by declines in the percentage with 3 or more family members and increases in older adults with 1–2 family members. Because of non-linear associations between family size and the measures of care gaps in this study, shifts in family size will not have a substantial influence on such gaps through 2040. Finally, the proportion of older adults with stepchildren, although substantial (40%), is stable across the projection period and does not have a strong association with lacking family caregivers or unmet need.

Our findings mirror studies that have projected, in a cross-national context, declines in family size among older adults (Alburez-Gutierrez et al., 2023). Yet, cross-national studies have documented wide variation in kinlessness (Verdery et al., 2019; Pittavino et al., 2024); the prevalence of care gaps (Wu et al., 2023); the role of non-kin among those aging alone (Mair, 2019); and whether aging without close kin is a marker of privilege or risk (Mair, 2024). Additional studies are needed to better understand how the pace of population aging, the degree of decline in family size and rise in kinlessness, and the strength of the association between family and care patterns as reflected in norms and policies about family and care are likely to influence future numbers of older adults with care gaps.

### Limitations

This study has several limitations. First, the main projections in our analysis are limited to U.S. born single-race White and Black older adults because of lack of availability of historical data necessary to generate reliable kinship simulations for Hispanic and other groups. Although 80% of the older population is represented by these two groups in 2022, immigrant and other U.S.-born racial and ethnic groups may grow as a percentage of the older population through 2030 and later (Colby & Ortman, 2014). Nevertheless, based on alternative projections that included these groups, assuming their growth would be on par with the rest of the population, we conclude that the main findings of the study are not altered.

Second, the simulations do not consider alternatives to marriage in later life such as cohabitation, which has grown substantially in later life in recent decades, reaching 15% among the 65 and older age group in 2023 (Gokhale & Smetters, 2025). Nor did we explore shifts in availability of family members other than spouses and children. Accordingly, older adults that we have categorized as having no spouse or children may increasingly have other close relationships in their safety net.

Third, our results apply current care patterns to expected changes in family structure that are driven by ongoing demographic change. Thus, we assumed the probability of needing care will remain constant in the future by family structure and size. In the past decade, care needs generally have been stable or declined for older adults (Freedman & Cornman, 2023); yet, increases in activity limitations that have been documented at younger ages and in midlife (Zajacova & Margolis, 2024; Martin et al., 2010) call into question the direction of future trends in later-life care needs. Activity limitation patterns in the future could interact with the evolution of family structure in ways not captured in the current analysis.

Fourth, although we projected both the size and structure of the care network and unmet need for care, both of which are related to intensity of care, we did not project the future frequency or hours of care. Intensity of care needs may be influenced by changes in the profile of chronic conditions experienced by older adults and the environments in which activities take place. Moreover, the intensity of care provided by family caregivers may be influenced by competing time demands from work or other family members. With respect to work, the percentage of employed caregivers in the United States has remained stable at about 40% (Wolff et al., 2025), but continued increases in labor force participation by women (Bureau of Labor Statistics, 2025) and in caregiving participation by men (Wolff et al., 2025) could lead to more caregivers facing dual demands of work and care in the future. Such trends could lead to less time available by family members for care, if workplace supports such as paid caregiving leave policies and flexible and remote work arrangements do not keep pace (Jacobs, 2020).

Finally, although there were scientific benefits to considering the contribution of only one factor at a time (e.g. age structure, family structure, family size), sample size limitations precluded our ability to examine all factors simultaneously. Nor did we jointly project numbers with no family caregivers and unmet care needs. We also examined only a limited number of potentially important population subgroups. As with any projection, macro-level influences, such as changes to payment policies, could affect the receipt of care and adequacy of support. Consequently, our projections should be considered hypothetical comparisons based on the current policy and social environment and expected demographic change rather than forecasts of future U.S. experience.

### Conclusion

The findings in this paper enhance our understanding of the implications of the ongoing shifts in family structure and size among for addressing care needs of U.S. older adults. Family care networks may be marginally smaller in the future, as fewer family members are available to address care needs. Smaller networks may face fewer conflicts and disagreements in meeting the care needs of an older relative, but time demands for those providing assistance may increase. Enhanced access to supportive services, training for family caregivers, job flexibility for those who work, and better integration with the healthcare system have been proposed as key avenues for strengthening family care and supporting caregivers (National Family Caregiver Strategy, 2022). These strategies will only grow in importance as care networks continue to evolve.

Predictions that shifts in family structure and size will impose only small increases in those with no family caregivers—including those relying only on the paid care market—is a welcome finding. Yet, all else equal, age structure shifts will put upward pressure on the demand for paid care. Home health and personal care aides are among the fastest growing occupations in the United States. (Bureau of Labor Statistics, 2022); but even though use of paid care has increased (Van Houtven et al., 2020), the home-based workforce has not kept pace (Kreider & Werner, 2023). Moreover, although most older adults with care needs prefer to receive assistance at home (Kasper et al., 2019), access to professional home-based care is often limited to economically advantaged groups and to those eligible for public programs such as Medicaid, which varies substantially across states (Friedman et al., 2021; Johnson & Wang, 2019). Prior studies have documented elevated rates of unmet need among those receiving paid care services (Morales & Robert, 2022), reinforcing calls to improve training and modify state delegation laws to better integrate paid aides into care teams (Stone & Bryant, 2019).

Our study adds to calls for policy attention to the millions of older adults experiencing unmet need and those who care for them (Li et al., 2023; Werner & Konetzka, 2022; Administration for Community Living, 2022). 15% of older adults—more than half of those with care needs—have experienced a recent adverse consequence reflecting an unmet care need. As a consequence of population growth and aging, the United States can expect to have 16 million older adults with unmet care needs by 2040, many of whom will have no family caregivers. Attention should focus on research to develop strategies to increase availability and accessibility of high-quality paid care; to support family and friends who may step into and stay in the caregiving role; and to engage medical professionals in identifying older adults at risk for care gaps. More generally, policy solutions aimed at reshaping paid long-term care options (Werner & Kotezka, 2022) and providing supports to family caregivers (Administration for Community Living, 2022) are essential—not because of decays in family life—but because of the sizeable gaps in the current patch work and piece meal system of caring for older adults in the United States.

## Supplementary Information

Below is the link to the electronic supplementary material.

Supplementary Material 1 (PDF 775 KB)

## Data Availability

The National Health and Aging Trends Study data files are made available through nhats.org. Code to produce the data files for the projections in this manuscript is available online from https://osf.io/z3suy/?view_only=dac305f99c414e578aceb0eb3aadf5cd.

## References

[CR2] Administration for Community Living (2022). 2022 *National strategy to support family caregivers*. Washington DC: U.S. Department of Health and Human Services. https://acl.gov/CaregiverStrategy

[CR1] Alburez-Gutierrez, D., Williams, I., & Caswell, H. (2023). Projections of human kinship for all countries. *Proceedings of the National Academy of Sciences*, *120*(52), e2315722120. 10.1073/pnas.2315722120 10.1073/pnas.2315722120 PMC10756196 38113253

[CR3] Beach, S. R., & Schulz, R. (2017). Family caregiver factors associated with unmet needs for care of older adults. *Journal of the American Geriatrics Society*, *65*(3), 560–566. 10.1111/jgs.14547 27935019 10.1111/jgs.14547

[CR5] Bureau of Labor Statistics (2022). *Employment projections. Table 1.3: Fastest growing occupations, 2021 and projected 2031*. U.S. Department of Labor. https://www.bls.gov/emp/tables/fastest-growing-occupations.htm

[CR6] Bureau of Labor Statistics (2025). *Employment status of the civilian noninstitutional population 16 years and over by sex, last 40 years*. U.S. Department of Labor. https://www.bls.gov/cps/cpsaat02.htm

[CR38] U.S. Census Bureau (2017). *2017 National Projections*. https://www.census.gov/data/tables/2017/demo/popproj/2017-summary-tables.html

[CR7] Colby, S. L., & Ortman, J. M. (2014). Projections of the size and composition of the U.S. population: 2014 to 2060. *Current Population Reports*, P25-1143, U.S. Census Bureau. https://mronline.org/wp-content/uploads/2019/08/p25-1143.pdf

[CR10] Freedman, V. A. (2018). The demography of late-life disability. In National Academies of Sciences, Engineering, and Medicine. *Future directions for the demography of aging: Proceedings of a Workshop* (pp.269–307). The National Academies Press. 10.17226/25064 29989766

[CR12] Freedman, V. A., & Cornman, J. C. (2023). *National Health and Aging Trends Study trends chart book: Key trends, measures and detailed Tables 2011–2021*. National Health and Aging Trends Study. micda.isr.umich.edu/research/nhats-trends-dashboards

[CR100] Freedman, V. A., & Spillman, B. C. (2014). Disability and care needs among older Americans. *Milbank Memorial Quarterly, 92*(3), 509–541. 10.1111/1468-0009.12076 10.1111/1468-0009.12076 PMC4221755 25199898

[CR8] Freedman, V. A., & Kasper, J. D. (2019). Cohort profile: The National Health and Aging Trends Study (NHATS). *International Journal of Epidemiology*, *48*(4), 1044–1045g. 10.1093/ije/dyz109 31237935 10.1093/ije/dyz109 PMC6934030

[CR9] Freedman, V. A., Schrack, J. A., & Skehan, M. E. (2024). *National Health and Aging Trends Study user guide: Rounds 1–13 final release*. Johns Hopkins Bloomberg School of Public Health. www.NHATS.org.

[CR13] Friedman, E. M., Ghosh-Dastidar, M., Ruder, T., Siconolfi, D., & Shih, R. A. (2021). Trends In home care versus nursing home workforce sizes: Are states converging or diverging over time? *Health Affairs*, *40*(12), 1875–1882. 10.1377/hlthaff.2021.00749 34871084 10.1377/hlthaff.2021.00749 PMC10880546

[CR14] Gokhale, J., & Smetters, K. (2025). *Change in American families: Favoring cohabitation over marriage.* https://budgetmodel.wharton.upenn.edu/issues/2025/2/19/change-in-american-families-favoring-cohabitation-over-marriage

[CR15] Hass, Z., DePalma, G., Craig, B. A., Xu, H., & Sands, L. P. (2017). Unmet need for help with activities of daily living disabilities and emergency department admissions among older Medicare recipients. *The Gerontologist*, *57*(2), 206–210. 10.1093/geront/gnv142 26603181 10.1093/geront/gnv142 PMC5881665

[CR16] He, S., Craig, B. A., Xu, H., Covinsky, K. E., Stallard, E., Thomas, J. 3rd, Hass, Z., & Sands, L. P. (2015). Unmet need for ADL assistance is associated with mortality among older adults with mild disability. *Journals of Gerontology*, *70*(9), 1128–1132. 10.1093/gerona/glv028 10.1093/gerona/glv028 PMC4841172 25834196

[CR17] Jacobs, E. (2020). Family caregiving, caregiving leave, and labor market outcomes. In I. Sawhill & B. Stevenson (Eds.), *Paid leave for caregiving: Issues and answers* (pp. 31–65). AEI/Brookings. https://www.aei.org/wp-content/uploads/2020/11/Paid-Leave-for-Caregiving.pdf

[CR18] Johnson, R., & Wang, C. (2019). How many older adults can afford to purchase home care? *ASPE Research Brief*. Department of Health and Human Services. https://aspe.hhs.gov/reports/how-many-older-adults-can-afford-purchase-home-care

[CR20] Kasper, J. D., Wolff, J. L., & Skehan, M. (2019). Care arrangements of older adults: What they prefer, what they have, and implications for quality of life. *The Gerontologist*, *59*(5), 845–855. 10.1093/geront/gny127 30476072 10.1093/geront/gny127 PMC6857686

[CR21] Kreider, A. R., & Werner, R. M. (2023). The home care workforce has not kept pace with growth in home and community-based services. *Health Affairs*, *42*(5), 650–657. 10.1377/hlthaff.2022.01351 37075251 10.1377/hlthaff.2022.01351 PMC10278236

[CR19] Li, J., Ha, J., & Hoffman, G. (2023). Unaddressed functional difficulty and care support among White, Black, and Hispanic older adults in the last decade. *Health Affairs Scholar*, *1*(3). 10.1093/haschl/qxad041 10.1093/haschl/qxad041 PMC10809881 38274860

[CR23] Lin, I. F., & Wolf, D. A. (2022). Division of parent care among adult children. *Journals of Gerontology*, *75*(10), 2230–2239. 10.1093/geronb/gbz162 10.1093/geronb/gbz162 PMC7751154 31883022

[CR22] Lin, I. F., Brown, S. L., & Cupka, C. J. (2018). A national portrait of stepfamilies in later life. *Journals of Gerontology*, *73*(6), 1043–1054. 10.1093/geronb/gbx150 10.1093/geronb/gbx150 PMC6093452 29190365

[CR24] Mair, C. A. (2019). Alternatives to aging alone? Kinlessness and the importance of friends across European contexts. *Journals of Gerontology*, *74*(8), 1416–1428. 10.1093/geronb/gbz029 10.1093/geronb/gbz029 PMC6777764 30855074

[CR25] Mair, C. A. (2024). Successfully aging alone? Unequal global opportunities and rising risks in family-based models of care cross-nationally. *The Gerontologist*, *65*(1), gnae104. 10.1093/geront/gnae104 39126221 10.1093/geront/gnae104

[CR26] Margolis, R., & Verdery, A. M. (2017). Older adults without close kin in the United States. *Journals of Gerontology*, *72*(4), 688–693. 10.1093/geronb/gbx068 10.1093/geronb/gbx068 PMC5927096 28575387

[CR27] Martin, L. G., Freedman, V. A., Schoeni, R. F., & Andreski, P. M. (2010). Trends in disability and related chronic conditions among people ages fifty to sixty-four. *Health Affairs*, *29*(4), 725–731. 10.1377/hlthaff.2008.0746 20368601 10.1377/hlthaff.2008.0746 PMC2874878

[CR29] Morales, M. J., & Robert, A. S. (2022). Examining consequences related to unmet care needs across the long-term care continuum. *Journals of Gerontology*, *77*(Supplement 1), S63–73. 10.1093/geronb/gbab210 10.1093/geronb/gbab210 PMC9122632 35030256

[CR31] National Family Caregiver Strategy (2022). Administration for Community Living. https://acl.gov/CaregiverStrategy

[CR30] National Academies of Sciences, Engineering, and Medicine. (2016). *Families Caring for an Aging America*. National Academies. 10.17226/23606 27905704

[CR32] Patterson, S. E., & Freedman, V. A. (2025). Family structure and unmet care needs among older adults with and without dementia in the U.S. *The Gerontologist*, *65*(5), gnaf062. 10.1093/geront/gnaf062 39932438 10.1093/geront/gnaf062 PMC12048798

[CR33] Patterson, S. E., Schoeni, R. F., Freedman, V. A., & Seltzer, J. A. (2022). Care received and unmet care needs among older parents in biological and step families. *Journals of Gerontology*, *77*(Suppl_1), S51–S62. 10.1093/geronb/gbab178 10.1093/geronb/gbab178 PMC9122660 34893839

[CR34] Pittavino, M., Arpino, B., & Pirani, E. (2024). Kinlessness at older ages: Prevalence and heterogeneity in 27 countries. *Journals of Gerontology*, *80*(1), gbae180. 10.1093/geronb/gbae180 10.1093/geronb/gbae180 PMC11662209 39470404

[CR35] Redfoot, D., Feinberg, L., & Houser, A. N. (2013). *The aging of the baby boom and the growing care gap: A look at future declines in the availability of family caregivers*. *AARP Public Policy Institute*. https://www.caregiver.org/uploads/2023/02/baby-boom-and-the-growing-care-gap-insight-AARP-ppi-ltc.pdf

[CR36] Schoeni, R. F., Freedman, V. A., Cornman, J. C., & Seltzer, J. A. (2022). The strength of parent-adult child ties in biological families and stepfamilies: evidence from time diaries from older aAdults. *Demography*, *59*(5), 1821–1842. 10.1215/00703370-10177468 36112392 10.1215/00703370-10177468 PMC9930742

[CR37] Stone, R. I., & Bryant, N. S. (2019). The future of the home care workforce: Training and supporting aides as members of home-based care teams. *Journal of the American Geriatrics Society*, *67*(S2), S444–S448. 10.1111/jgs.15846 31074856 10.1111/jgs.15846

[CR39] Van Houtven, C. H., Konetzka, R. T., Taggert, E., & Coe, N. B. (2020). Informal and formal home care for older adults with disabilities increased, 2004–16. *Health Affairs*, *39*(8), 1297–1301. 10.1377/hlthaff.2019.01800 32744936 10.1377/hlthaff.2019.01800 PMC8729158

[CR40] Verdery, A., & Margolis, R. (2017). Projections of white and black older adults without living kin in the United States, 2015 to 2060. *Proceedings of the National Academies of Sciences, 114*(42), 11109–11114. 10.1073/pnas.1710341114 10.1073/pnas.1710341114 PMC5651770 28973934

[CR41] Verdery, A. M., Margolis, R., Zhou, Z., Chai, X., & Rittirong, J. (2019). Kinlessness around the world. *Journals of Gerontology*, *74*(8), 1394–1405. 10.1093/geronb/gby138 10.1093/geronb/gby138 PMC6777763 30423167

[CR42] Verdery, A. M., Smith-Greenaway, E., Margolis, R., & Daw, J. (2020). Tracking the reach of COVID-19 kin loss with a bereavement multiplier applied to the United States. *Proceedings of the National Academy of Sciences*, *117*(30), 17695–17701. 10.1073/pnas.2007476117 10.1073/pnas.2007476117 PMC7395491 32651279

[CR43] Werner, R. M., & Konetzka, R. T. (2022). Reimagining financing and payment of long-term care. *Journal of the American Medical Director’s Association, 23*(2), 220–224. 10.1016/j.jamda.2021.11.030 10.1016/j.jamda.2021.11.030 PMC8695540 34942158

[CR11] Wolff, J. L. (2020). The changing landscape of family caregiving in the United States. *Paid leave for caregiving: Issues and answers* (pp. 11–30). AEI/Brookings. https://www.aei.org/wp-content/uploads/2020/11/Paid-Leave-for-Caregiving.pdf

[CR44] Wolff, J. L., Cornman, J. C., & Freedman, V. A. (2025). The number of family caregivers helping older US adults increased from 18 million to 24 million, 2011-22. *Health Affairs*, *44*(2), 187–195. 10.1377/hlthaff.2024.00978 39899774 10.1377/hlthaff.2024.00978 PMC11869104

[CR45] Wu, H., Sheftel, M. R., M. G., & Verdery, A. M. (2023). The care gap in later life across European countries. *Journals of Gerontology*, *78*(11), 1935–1946. 10.1093/geronb/gbad118 10.1093/geronb/gbad118 PMC10645305 37589455

[CR46] Wu, H., Margolis, R., Verdey, A. M., & Patterson, S. E. (2024). Changes in family structure and increasing care gaps in the United States, 2015–2050. *Demography*, *61*(5), 1403–1426. 10.1215/00703370-11551558 39259138 10.1215/00703370-11551558 PMC11629368

[CR47] Xu, H., Covinsky, K. E., Stallard, E., Thomas, J. 3rd, & Sands, L. P. (2012). Insufficient help for activity of daily living disabilities and risk of all-cause hospitalization. *Journal of the American Geriatrics Society*, *60*(5), 927–933. 10.1111/j.1532-5415.2012.03926.x 22587855 10.1111/j.1532-5415.2012.03926.x PMC4162318

[CR48] Zajacova, A., & Margolis, R. (2024). Trends in disability and limitations among U.S. adults age 18–44, 2000–2018. *American Journal of Epidemiology*, *194*(5), 1381–1388. 10.1093/aje/kwae262 10.1093/aje/kwae262 PMC12055464 39136389

