# Supplementary material for: The Future Availability of Family Caregivers: Implications for Late-Life Care Gaps
Source: Popul Res Policy Rev. 2026 Jun 1;45(3):33. doi: 10.1007/s11113-026-10016-4 (PMC13226366; doi:10.1007/s11113-026-10016-4)
Supplement: Supplementary file 1 — Supplementary Material 1 (PDF 775 KB) [file 11113_2026_10016_MOESM1_ESM.pdf]

ONLINE SUPPLEMENT

The Future Availability of Family Caregivers: Implications for Late-Life Care Gaps

Vicki A. Freedman
Rachel Margolis
Ashton M. Verdery
Emily M. Agree &
Esther M. Friedman

Freedman, V., Margolis, R., Verdery, A. et al. The Future Availability of Family Caregivers: Implications for Late-Life Care Gaps. Popul Res Policy Rev **45**, 33 (2026). https://doi.org/10.1007/s11113-026-10016-4

Table S1. Probability of Having Family or Unpaid Caregivers and Probability of Having Unmet Need by Family Structure and Size, 2022 National Health and Aging Trends Study, U.S. Population Ages 65 and Older, Before and After Recalibration to Population Projections

			Probability of having Family or Unpaid Caregivers						Probability of having Unmet Need		
	Weighted %	Probability of having a care need[a]	No care need[a]	Zero caregivers	Only paid caregivers	1	2	≥3	No care need[a]	No unmet need	Unmet need
					After Recalibration						
Population: All											
Family network structure[b]											
No spouse											
…no biological children or stepchildren	5.0%	0.3494	0.6506	0.1330	0.0395	0.1300	0.0269	0.0201	0.6506	0.1472	0.2022
…has biological children only	20.7%	0.3866	0.6134	0.1052	0.0225	0.1298	0.0790	0.0500	0.6134	0.1719	0.2146
…has any stepchildren	16.3%	0.3497	0.6503	0.1046	0.0297	0.1173	0.0603	0.0378	0.6503	0.1581	0.1917
Has spouse											
…no biological or stepchildren	5.0%	0.1933	0.8067	0.1087	0.0000	0.0522	0.0262	0.0061	0.8067	0.1054	0.0878
…has biological children only	29.3%	0.2082	0.7918	0.0741	0.0015	0.0881	0.0271	0.0174	0.7918	0.1065	0.1017
…has any stepchildren	23.8%	0.2518	0.7482	0.1021	0.0045	0.0983	0.0288	0.0182	0.7482	0.1192	0.1326
Family size[b]											
0	5.0%	0.3494	0.6506	0.1330	0.0395	0.1300	0.0269	0.0201	0.6506	0.1472	0.2022
1	10.0%	0.2729	0.7271	0.0967	0.0044	0.1082	0.0370	0.0266	0.7271	0.1194	0.1535
2	15.4%	0.3045	0.6955	0.1104	0.0195	0.1003	0.0495	0.0248	0.6955	0.1323	0.1722
3	19.7%	0.2473	0.7527	0.0763	0.0084	0.0927	0.0488	0.0211	0.7527	0.1203	0.1270
≥4	49.8%	0.2894	0.7106	0.0972	0.0118	0.1066	0.0426	0.0312	0.7106	0.1404	0.1490
					Before Recalibration						
Family network structure[b]											
No spouse											
…no biological children or stepchildren	8.2%	0.3494	0.6506	0.1330	0.0395	0.1300	0.0269	0.0201	0.6506	0.1472	0.2022
…has biological children only	31.9%	0.3866	0.6134	0.1052	0.0225	0.1298	0.0790	0.0500	0.6134	0.1719	0.2146
…has any stepchildren	4.8%	0.2641	0.7359	0.0843	0.0401	0.0797	0.0388	0.0213	0.7359	0.1329	0.1312
Has spouse											
…no biological or stepchildren	5.0%	0.1933	0.8067	0.1087	0.0000	0.0522	0.0262	0.0061	0.8067	0.1054	0.0878
…has biological children only	38.3%	0.2082	0.7918	0.0741	0.0015	0.0881	0.0271	0.0174	0.7918	0.1065	0.1017
…has any stepchildren	11.9%	0.2607	0.7393	0.1228	0.0005	0.1013	0.0222	0.0138	0.7393	0.1192	0.1415
Family size[b]											
0	8.2%	0.3494	0.6506	0.1330	0.0395	0.1300	0.0269	0.0201	0.6506	0.1472	0.2022
1	12.3%	0.2729	0.7271	0.0967	0.0044	0.1082	0.0370	0.0266	0.7271	0.1194	0.1535
2	19.3%	0.3045	0.6955	0.1104	0.0195	0.1003	0.0495	0.0248	0.6955	0.1323	0.1722
3	28.3%	0.2473	0.7527	0.0763	0.0084	0.0927	0.0488	0.0211	0.7527	0.1203	0.1270
≥4	32.0%	0.2940	0.7060	0.0976	0.0095	0.1086	0.0421	0.0361	0.7060	0.1475	0.1465

Note: Probabilities exclude foreign born and Hispanic older adults and racial groups other than White, Black.
[a]Care need defined as gets help with self-care or mobility or with household activities for health/functioning reasons or has an unmet need for self-care, mobility or household activity assistance. [b]Count of spouse, biological children and stepchildren.

Table S2. Probability of Having Family or Unpaid Caregivers and Probability of Having Unmet Need by Age, Gender, Race, and Family Characteristics, U.S.-Born White and Black Adults Ages 65 and Older: 2022 National Health and Aging Trends Study

	Probability of Having Caregivers, by Type and Number						Probability of having Unmet Need		
	No care need[a]	Zero caregivers	Only paid caregivers	1 family or unpaid caregiver	2 family or unpaid caregivers	≥ 3 family or unpaid caregivers	No care need[a]	No unmet need	Unmet need
Population: All									
Overall	0.7152	0.0968	0.0130	0.1042	0.0436	0.0272	0.7152	0.1334	0.1514
Age									
65–74	0.7745	0.0827	0.0108	0.0921	0.0241	0.0158	0.7745	0.0939	0.1315
75–84	0.7017	0.1210	0.0079	0.1003	0.0405	0.0286	0.7017	0.1388	0.1596
≥85	0.4544	0.0930	0.0396	0.1787	0.1527	0.0816	0.4544	0.3187	0.2269
Gender									
Men	0.7532	0.0977	0.0132	0.0915	0.0273	0.0171	0.7532	0.1188	0.1280
Women	0.6849	0.0962	0.0127	0.1143	0.0565	0.0353	0.6849	0.1451	0.1700
Race									
White	0.7265	0.0970	0.0122	0.1016	0.0390	0.0237	0.7265	0.1269	0.1466
Black	0.6191	0.0956	0.0199	0.1284	0.0855	0.0598	0.6191	0.1937	0.1873
Family structure[b]									
No spouse									
…no biological children or stepchildren	0.6506	0.1330	0.0395	0.1300	0.0269	0.0201	0.6506	0.1472	0.2022
…has biological children only	0.6134	0.1052	0.0225	0.1298	0.0790	0.0500	0.6134	0.1719	0.2146
…has any stepchildren	0.6503	0.1046	0.0297	0.1173	0.0603	0.0378	0.6503	0.1581	0.1917
Has spouse									
…no biological children or stepchildren	0.8067	0.1087	0.0000	0.0522	0.0262	0.0061	0.8067	0.1054	0.0878
…has biological children only	0.7918	0.0741	0.0015	0.0881	0.0271	0.0174	0.7918	0.1065	0.1017
…has any stepchildren	0.7482	0.1021	0.0045	0.0983	0.0288	0.0182	0.7482	0.1192	0.1326
Family size[b,c]									
0	0.6506	0.1330	0.0395	0.1300	0.0269	0.0201	0.6506	0.1472	0.2022
1	0.7271	0.0967	0.0044	0.1082	0.0370	0.0266	0.7271	0.1194	0.1535
2	0.6955	0.1104	0.0195	0.1003	0.0495	0.0248	0.6955	0.1323	0.1722
3	0.7527	0.0763	0.0084	0.0927	0.0488	0.0211	0.7527	0.1203	0.1270
≥4	0.7060	0.0976	0.0095	0.1086	0.0421	0.0361	0.7060	0.1475	0.1465

Note: Probabilities exclude foreign born and Hispanic older adults and racial groups other than White, Black.
[a]Care need defined as gets help with self-care or mobility or with household activities for health/functioning reasons or has an unmet need for self-care, mobility or household activity assistance. [b]Family structure and size estimates realigned to match population projections. See text for details. [c]Count of spouse, biological children and stepchildren.

Table S3. Probability of Having Family or Unpaid Caregivers and Probability of Having Unmet Need by Age and Family Characteristics, Excluded Cases: 2022 National Health and Aging Trends Study

		Probability of Having Caregivers, by Type and Number						Probability of Having Unmet Need		
	weighted %	No care need[a]	Zero caregivers	Only paid caregivers	1 family or unpaid caregiver	2 family or unpaid caregivers	≥ 3 family or unpaid caregivers	No care need[a]	No unmet need	Unmet need
Overall	100.0	0.6729	0.0852	0.0147	0.1278	0.0552	0.0441	0.6729	0.1408	0.1863
Age										
65–74	59.8	0.7591	0.0736	0.0036	0.1104	0.0344	0.0188	0.7591	0.0911	0.1498
75–84	29.9	0.6046	0.1034	0.0288	0.1225	0.0661	0.0715	0.6046	0.1815	0.2139
≥85	10.3	0.3615	0.0916	0.0388	0.2479	0.1469	0.1132	0.3615	0.3164	0.3221
Family structure[b]										
No spouse										
…no biological children or stepchildren	5.0%	0.6034	0.1118	0.0403	0.1339	0.1107	0.0000	0.6034	0.1482	0.2484
…has biological children only	20.7%	0.5966	0.0774	0.0283	0.1545	0.0692	0.0740	0.5966	0.1598	0.2436
…has any stepchildren	16.3%	0.5799	0.0933	0.0322	0.1574	0.0723	0.0648	0.5799	0.1793	0.2407
Has spouse										
…no biological children or stepchildren	4.0%	0.5536	0.1764	0.0000	0.2513	0.0186	0.0000	0.5536	0.1549	0.2915
…has biological children only	29.3%	0.7536	0.0714	0.0000	0.0944	0.0444	0.0362	0.7536	0.1163	0.1301
…has any stepchildren	24.8%	0.7345	0.0827	0.0067	0.1048	0.0400	0.0312	0.7345	0.1246	0.1408
Family size[b,c]										
0	5.0%	0.6034	0.1118	0.0403	0.1339	0.1107	0.0000	0.6034	0.1482	0.2484
1	10.1%	0.6502	0.0916	0.0081	0.2138	0.0290	0.0073	0.6502	0.1398	0.2034
2	15.4%	0.7064	0.0781	0.0351	0.0925	0.0330	0.0548	0.7064	0.1123	0.1813
3	19.7%	0.7011	0.0810	0.0086	0.1136	0.0361	0.0596	0.7011	0.1384	0.1605
≥4	49.8%	0.6617	0.0850	0.0096	0.1264	0.0693	0.0479	0.6617	0.1498	0.1881

Note: Excluded cases include foreign born, Hispanic, and racial groups other than White, Black.
[a]Care need defined as gets help with self-care or mobility or with household activities for health/functioning reasons or has an unmet need for self-care, mobility or household activity assistance. [b]Family structure and size estimates realigned to match population projections. See text for details. [c]Count of spouse, biological children and stepchildren.

Table S4. Probability of Having Family or Unpaid Caregivers and Probability of Having Unmet Need by Age, Race, and Family Characteristics, U.S.-Born White and Black Adults Ages 65 and Older Subgroups: 2022 National Health and Aging Trends Study

	Probability of Having Caregivers, by Type and Number						Probability of having Unmet Need		
	No care need[a]	Zero caregivers	Only paid caregivers	1 family or unpaid caregiver	2 family or unpaid caregivers	≥ 3 family or unpaid caregivers	No care need[a]	No unmet need	Unmet need
Population: Men									
Overall	0.7532	0.0977	0.0132	0.0915	0.0273	0.0171	0.7532	0.1188	0.1280
Age									
65–74	0.8013	0.0693	0.0130	0.0894	0.0189	0.0080	0.8013	0.0814	0.1172
75–84	0.7216	0.1433	0.0025	0.0809	0.0313	0.0204	0.7216	0.1425	0.1359
≥85	0.5722	0.1004	0.0555	0.1457	0.0647	0.0615	0.5722	0.2629	0.1649
Race									
White	0.7606	0.0999	0.0123	0.0874	0.0249	0.0149	0.7606	0.1114	0.1280
Black	0.6801	0.0756	0.0220	0.1322	0.0509	0.0393	0.6801	0.1918	0.1281
Family structure[b]									
No spouse									
…no biological children or stepchildren	0.7008	0.0979	0.0203	0.1498	0.0139	0.0173	0.7008	0.1360	0.1632
…has biological children only	0.6796	0.1022	0.0315	0.1119	0.0390	0.0358	0.6796	0.1313	0.1891
…has any stepchildren	0.6677	0.1122	0.0438	0.1134	0.0319	0.0312	0.6677	0.1461	0.1862
Has spouse									
…no biological children or stepchildren	0.8181	0.1024	0.0000	0.0526	0.0258	0.0011	0.8181	0.1217	0.0602
…has biological children only	0.8144	0.0678	0.0022	0.0764	0.0255	0.0137	0.8144	0.1027	0.0830
…has any stepchildren	0.7367	0.1261	0.0083	0.0931	0.0248	0.0110	0.7367	0.1191	0.1441
Family size[b,c]									
0	0.7008	0.0979	0.0203	0.1498	0.0139	0.0173	0.7008	0.1360	0.1632
1	0.7739	0.0874	0.0019	0.0985	0.0264	0.0119	0.7739	0.1061	0.1198
2	0.7482	0.1075	0.0215	0.0707	0.0367	0.0154	0.7482	0.1039	0.1478
3	0.7764	0.0803	0.0112	0.0880	0.0344	0.0098	0.7764	0.1086	0.1150
≥4	0.7467	0.1032	0.0133	0.0923	0.0236	0.0210	0.7467	0.1269	0.1264

Table S4 (Continued)

Population: Women									
Overall	0.6849	0.0962	0.0127	0.1143	0.0565	0.0353	0.6849	0.1451	0.1700
Age									
65–74	0.7523	0.0939	0.0090	0.0942	0.0284	0.0222	0.7523	0.1043	0.1434
75–84	0.6852	0.1026	0.0124	0.1163	0.0482	0.0353	0.6852	0.1357	0.1791
≥85	0.3856	0.0887	0.0303	0.1979	0.2041	0.0933	0.3856	0.3513	0.2631
Race									
White	0.6990	0.0946	0.0121	0.1130	0.0504	0.0308	0.6990	0.1394	0.1616
Black	0.5612	0.1100	0.0184	0.1256	0.1103	0.0745	0.5612	0.1950	0.2438
Family structure[b]									
No spouse									
…no biological children or stepchildren	0.6100	0.1612	0.0550	0.1141	0.0373	0.0223	0.6100	0.1563	0.2337
…has biological children only	0.5850	0.1065	0.0187	0.1375	0.0962	0.0561	0.5850	0.1894	0.2256
…has any stepchildren	0.6483	0.0973	0.0204	0.1193	0.0740	0.0406	0.6483	0.1614	0.1903
Has spouse									
…no biological children or stepchildren	0.7904	0.1178	0.0000	0.0516	0.0269	0.0134	0.7904	0.0820	0.1276
…has biological children only	0.7676	0.0809	0.0006	0.1007	0.0288	0.0214	0.7676	0.1107	0.1217
…has any stepchildren	0.7626	0.0763	0.0016	0.1052	0.0287	0.0256	0.7626	0.1171	0.1203
Family size[b,c]									
0	0.6100	0.1612	0.0550	0.1141	0.0373	0.0223	0.6100	0.1563	0.2337
1	0.6967	0.1028	0.0062	0.1144	0.0439	0.0361	0.6967	0.1280	0.1753
2	0.6638	0.1121	0.0184	0.1181	0.0572	0.0305	0.6638	0.1494	0.1868
3	0.7309	0.0727	0.0057	0.0971	0.0621	0.0315	0.7309	0.1311	0.1380
≥4	0.6793	0.0909	0.0100	0.1205	0.0592	0.0402	0.6793	0.1521	0.1686

Table S4 (Continued)

Population: Married Adults									
Overall	0.7818	0.0877	0.0011	0.0877	0.0260	0.0156	0.7818	0.1092	0.1090
Age									
65–74	0.8272	0.0666	0.0003	0.0751	0.0203	0.0108	0.8272	0.0762	0.0966
75–84	0.7341	0.1243	0.0164	0.0942	0.0267	0.0204	0.7341	0.1406	0.1253
≥85	0.5795	0.1055	0.0000	0.1802	0.0795	0.0388	0.5795	0.2730	0.1474
Race									
White	0.7879	0.0864	0.0011	0.0869	0.0238	0.0139	0.7879	0.1046	0.1075
Black	0.6911	0.1068	0.0017	0.0998	0.0586	0.0420	0.6911	0.1777	0.1313
Family structure[b]									
Has spouse									
…no biological children or stepchildren	0.8067	0.1087	0.0000	0.0522	0.0262	0.0061	0.8067	0.1054	0.0878
…has biological children only	0.7918	0.0741	0.0015	0.0881	0.0271	0.0174	0.7918	0.1065	0.1017
…has any stepchildren	0.7583	0.0983	0.0010	0.0941	0.0328	0.0154	0.7583	0.1121	0.1211
Family size[b,c]									
1	0.8067	0.1087	0.0000	0.0522	0.0262	0.0061	0.8067	0.1054	0.0878
2	0.7549	0.0784	0.0000	0.1070	0.0398	0.0200	0.7549	0.0936	0.1515
3	0.8201	0.0648	0.0023	0.0770	0.0230	0.0127	0.8201	0.0968	0.0831
≥4	0.7692	0.0940	0.0011	0.0941	0.0240	0.0175	0.7692	0.1176	0.1132
Population: Unmarried Adults									
Overall	0.6333	0.1080	0.0275	0.1245	0.0652	0.0415	0.6333	0.1633	0.2034
Age									
65–74	0.6911	0.1083	0.0280	0.1190	0.0301	0.0236	0.6911	0.1220	0.1870
75–84	0.6655	0.1173	0.0164	0.1071	0.0560	0.0377	0.6655	0.1368	0.1977
≥85	0.3973	0.0873	0.0501	0.1780	0.1861	0.1011	0.3973	0.3395	0.2632
Race									
White	0.6442	0.1111	0.0271	0.1213	0.0594	0.0369	0.6442	0.1568	0.1990
Black	0.5674	0.0895	0.0297	0.1439	0.1001	0.0695	0.5674	0.2023	0.2303
Family structure[b]									
No spouse									
…no biological children or stepchildren	0.6506	0.1330	0.0395	0.1300	0.0269	0.0201	0.6506	0.1472	0.2022
…has biological children only	0.6134	0.1052	0.0225	0.1298	0.0790	0.0500	0.6134	0.1719	0.2146
…has any stepchildren	0.6533	0.1040	0.0302	0.1160	0.0594	0.0372	0.6533	0.1571	0.1895
Family size[b,c]									
0	0.6506	0.1330	0.0395	0.1300	0.0269	0.0201	0.6506	0.1472	0.2022
1	0.6723	0.0885	0.0074	0.1467	0.0445	0.0406	0.6723	0.1290	0.1987
2	0.6651	0.1267	0.0295	0.0969	0.0545	0.0273	0.6651	0.1521	0.1828
3	0.6051	0.1014	0.0216	0.1271	0.1052	0.0396	0.6051	0.1718	0.2230
≥4	0.6126	0.0995	0.0316	0.1293	0.0703	0.0567	0.6126	0.1805	0.2069

Table S4 (Continued)

Population: White Adults									
Overall	0.7265	0.0970	0.0122	0.1016	0.0390	0.0237	0.7265	0.1269	0.1466
Age									
65–74	0.7901	0.0811	0.0097	0.0885	0.0182	0.0123	0.7901	0.0846	0.1253
75–84	0.7119	0.1230	0.0069	0.0969	0.0359	0.0254	0.7119	0.1315	0.1565
≥85	0.4585	0.0939	0.0408	0.1809	0.1515	0.0745	0.4585	0.3211	0.2204
Family structure[b]									
No spouse									
…no biological children or stepchildren	0.6533	0.1409	0.0410	0.1283	0.0168	0.0197	0.6533	0.1391	0.2076
…has biological children only	0.8091	0.1069	0.0000	0.0523	0.0272	0.0045	0.8091	0.1017	0.0893
…has any stepchildren	0.6230	0.1061	0.0244	0.1278	0.0744	0.0443	0.6230	0.1676	0.2094
Has spouse									
…no biological children or stepchildren	0.6657	0.1085	0.0264	0.1120	0.0543	0.0332	0.6657	0.1495	0.1848
…has biological children only	0.7991	0.0724	0.0016	0.0870	0.0248	0.0151	0.7991	0.1019	0.0990
…has any stepchildren	0.7550	0.1027	0.0035	0.0973	0.0250	0.0165	0.7550	0.1136	0.1313
Family size[b,c]									
0	0.6533	0.1409	0.0410	0.1283	0.0168	0.0197	0.6533	0.1391	0.2076
1	0.7408	0.0928	0.0036	0.1061	0.0332	0.0234	0.7408	0.1126	0.1466
2	0.7045	0.1112	0.0204	0.0977	0.0458	0.0204	0.7045	0.1234	0.1721
3	0.7631	0.0773	0.0090	0.0868	0.0442	0.0196	0.7631	0.1140	0.1228
≥4	0.7228	0.0970	0.0098	0.1053	0.0381	0.0269	0.7228	0.1350	0.1421
Population: Black Adults									
Overall	0.6109	0.0956	0.0199	0.1284	0.0855	0.0598	0.6109	0.1937	0.1954
Age									
65–74	0.6485	0.0958	0.0196	0.1210	0.0716	0.0435	0.6485	0.1695	0.1821
75–84	0.5910	0.0991	0.0190	0.1369	0.0910	0.0630	0.5910	0.2169	0.1920
≥85	0.4059	0.0820	0.0250	0.1524	0.1677	0.1670	0.4059	0.2894	0.3048
Family structure[b]									
No spouse									
…no biological children or stepchildren	0.6321	0.0796	0.0299	0.1418	0.0941	0.0225	0.6321	0.2015	0.1664
…has biological children only	0.5594	0.1005	0.0121	0.1412	0.1048	0.0820	0.5594	0.1964	0.2442
…has any stepchildren	0.5633	0.0829	0.0427	0.1461	0.0975	0.0675	0.5633	0.2068	0.2299
Has spouse									
…no biological children or stepchildren	0.7444	0.1571	0.0000	0.0491	0.0000	0.0494	0.7444	0.2062	0.0494
…has biological children only	0.6708	0.1021	0.0000	0.1069	0.0644	0.0558	0.6708	0.1824	0.1468
…has any stepchildren	0.6557	0.0980	0.0169	0.1149	0.0725	0.0419	0.6557	0.1829	0.1614
Family size[b,c]									
0	0.6321	0.0796	0.0299	0.1418	0.0941	0.0225	0.6321	0.2015	0.1664
1	0.6094	0.1306	0.0113	0.1256	0.0695	0.0536	0.6094	0.1777	0.2130
2	0.6181	0.1029	0.0124	0.1227	0.0811	0.0629	0.6181	0.2090	0.1730
3	0.6181	0.0638	0.0000	0.1687	0.1082	0.0411	0.6181	0.2016	0.1802
≥4	0.6054	0.0989	0.0276	0.1176	0.0818	0.0687	0.6054	0.1894	0.2052

Note: Probabilities exclude foreign born and Hispanic older adults and racial groups other than White, Black.

[a]Care need defined as gets help with self-care or mobility or with household activities for health/functioning reasons or has an unmet need for self-care, mobility or household activity assistance. [b]Family structure and size estimates realigned to match population projections. See text for details. [c]Count of spouse, biological children and stepchildren.

Table S5. Projected U.S. Population Ages 65 and Older by Age, Race, and Kin Characteristics, 2022–2040, Men, Women, Married, Unmarried, White and Black Adults

	Number (Millions)			Percentage		
	2022	2030	2040	2022	2030	2040
Population: Men						
Overall	22.7	27.0	27.9	100.0%	100.0%	100.0%
Age						
65–74	13.5	14.6	12.3	59.6%	54.0%	44.2%
75–84	7.0	9.5	10.9	30.7%	35.0%	39.0%
≥85	2.2	3.0	4.7	9.7%	11.0%	16.9%
Race						
White	20.4	23.9	24.2	89.7%	88.4%	86.6%
Black	2.3	3.1	3.7	10.3%	11.6%	13.4%
Family network structure[a]						
No spouse						
…no biological children or stepchildren	0.9	1.3	1.5	4.1%	4.9%	5.3%
…has biological children only	2.8	3.1	3.4	12.4%	11.5%	12.0%
…has any stepchildren	2.7	3.2	3.3	12.1%	11.7%	11.8%
Has spouse						
…no biological children or stepchildren	1.3	1.9	1.8	5.8%	7.0%	6.4%
…has biological children only	8.3	9.6	9.8	36.4%	35.4%	35.2%
…has any stepchildren	6.6	8.0	8.1	29.2%	29.5%	29.2%
Family size[a,b]						
0	0.9	1.3	1.5	4.1%	4.9%	5.3%
1	2.1	2.9	2.9	9.0%	10.6%	10.5%
2	3.0	3.7	3.8	13.2%	13.5%	13.6%
3	4.4	5.1	5.1	19.3%	18.7%	18.3%
≥4	12.3	14.1	14.6	54.3%	52.2%	52.3%
Population: Women						
Overall	27.8	32.8	34.2	100.0%	100.0%	100.0%
Age						
65–74	15.3	16.3	13.5	54.9%	49.7%	39.7%
75–84	8.7	11.8	13.3	31.4%	35.8%	39.0%
≥85	3.8	4.8	7.3	13.7%	14.5%	21.4%
Race						
White	24.5	28.4	28.9	87.9%	86.6%	84.7%
Black	3.4	4.4	5.2	12.1%	13.4%	15.3%
Family network structure[a]						
No spouse						
…no biological children or stepchildren	1.6	2.1	2.4	5.7%	6.5%	7.0%
…has biological children only	7.6	8.4	8.7	27.4%	25.7%	25.5%
…has any stepchildren	5.5	6.3	7.0	19.7%	19.3%	20.4%
Has spouse						
…no biological children or stepchildren	1.3	1.8	1.7	4.6%	5.6%	5.0%
…has biological children only	6.5	7.8	7.9	23.5%	23.9%	23.2%
…has any stepchildren	5.3	6.3	6.5	19.1%	19.1%	19.0%
Family size[a,b]						
0	1.6	2.1	2.4	5.7%	6.5%	7.0%
1	3.0	4.1	4.3	10.8%	12.6%	12.6%
2	4.8	5.8	6.0	17.3%	17.6%	17.7%
3	5.6	6.6	6.7	20.1%	20.0%	19.5%
≥4	12.8	14.2	14.8	46.1%	43.4%	43.2%

Table S5 (Continued)

Population: Married Adults						
Overall	29.4	35.4	35.9	100.0%	100.0%	100.0%
Age						
65–74	19.7	21.6	18.6	67.1%	61.1%	52.0%
75–84	8.1	11.5	13.5	27.7%	32.6%	37.7%
≥85	1.5	2.2	3.7	5.2%	6.3%	10.4%
Race						
White	26.8	32.0	31.8	91.4%	90.4%	88.7%
Black	2.5	3.4	4.1	8.6%	9.6%	11.3%
Family network structure[a]						
Has spouse						
…no biological children or stepchildren	2.6	3.7	3.5	8.8%	10.6%	9.7%
…has biological children only	14.8	17.4	17.8	50.4%	49.2%	49.5%
…has any stepchildren	12.0	14.2	14.6	40.8%	40.3%	40.8%
Family size[a,b]						
1	2.6	3.7	3.5	8.8%	10.6%	9.7%
2	3.5	4.3	4.5	12.0%	12.1%	12.7%
3	6.2	7.2	7.2	21.0%	20.5%	20.1%
≥4	17.1	20.1	20.6	58.1%	56.8%	57.4%
Population: Unmarried Adults						
Overall	21.2	24.5	26.2	100.0%	100.0%	100.0%
Age						
65–74	9.1	9.3	7.3	43.1%	37.8%	27.7%
75–84	7.5	9.7	10.7	35.6%	39.6%	40.7%
≥85	4.5	5.5	8.3	21.2%	22.5%	31.6%
Race						
White	18.0	20.3	21.3	85.0%	83.1%	81.3%
Black	3.2	4.1	4.9	15.0%	16.9%	18.7%
Family network structure[a]						
No spouse						
…no biological children or stepchildren	2.5	3.4	3.9	11.9%	14.1%	14.8%
…has biological children only	10.4	11.5	12.1	49.3%	47.2%	46.0%
…has any stepchildren	8.2	9.5	10.3	38.8%	38.8%	39.2%
Family size[a,b]						
0	2.5	3.4	3.9	11.9%	14.1%	14.8%
1	2.5	3.3	3.7	11.6%	13.4%	14.3%
2	4.3	5.1	5.3	20.2%	21.0%	20.1%
3	3.8	4.4	4.6	17.9%	17.9%	17.4%
≥4	8.1	8.2	8.8	38.3%	33.7%	33.4%

Table S5 (Continued)

Population: White Adults						
Overall	44.8	52.3	53.1	100.0%	100.0%	100.0%
Age						
65–74	25.2	26.5	21.6	56.3%	50.7%	40.7%
75–84	14.1	18.8	20.9	31.5%	36.0%	39.3%
≥85	5.5	7.0	10.7	12.2%	13.4%	20.1%
Family network structure[a]						
No spouse						
…no biological children or stepchildren	2.2	3.0	3.3	4.9%	5.7%	6.2%
…has biological children only	2.4	3.5	3.2	5.4%	6.7%	6.1%
…has any stepchildren	9.0	9.8	10.1	20.1%	18.7%	19.0%
Has spouse						
…no biological children or stepchildren	6.8	7.6	8.0	15.1%	14.5%	15.0%
…has biological children only	13.9	16.2	16.4	31.0%	31.0%	30.9%
…has any stepchildren	10.5	12.2	12.2	23.4%	23.4%	22.9%
Family size[a,b]						
0	2.2	3.0	3.3	4.9%	5.7%	6.2%
1	4.6	6.3	6.3	10.2%	12.0%	11.9%
2	7.0	8.4	8.5	15.7%	16.0%	16.1%
3	9.1	10.4	10.4	20.2%	19.9%	19.5%
≥4	22.0	24.3	24.6	49.0%	46.4%	46.3%
Population: Black Adults						
Overall	5.7	7.5	9.0	100.0%	100.0%	100.0%
Age						
65–74	3.6	4.4	4.3	63.0%	58.0%	47.8%
75–84	1.6	2.4	3.3	27.3%	32.0%	37.1%
≥85	0.6	0.8	1.4	9.7%	10.0%	15.1%
Family network structure[a]						
No spouse						
…no biological children or stepchildren	0.3	0.5	0.6	5.3%	6.1%	6.5%
…has biological children only	0.2	0.2	0.3	2.7%	3.2%	2.9%
…has any stepchildren	1.4	1.8	2.0	25.3%	23.3%	22.1%
Has spouse						
…no biological children or stepchildren	1.4	1.9	2.3	25.2%	25.5%	26.1%
…has biological children only	0.9	1.2	1.4	15.9%	15.3%	15.1%
…has any stepchildren	1.5	2.0	2.4	25.7%	26.6%	27.3%
Family size[a,b]						
0	0.3	0.5	0.6	5.3%	6.1%	6.5%
1	0.5	0.7	0.9	8.6%	9.8%	10.0%
2	0.8	1.1	1.3	13.7%	14.2%	14.3%
3	0.9	1.2	1.4	15.8%	15.8%	15.8%
≥4	3.2	4.1	4.8	56.6%	54.2%	53.4%

Note: Projections exclude foreign born and Hispanic older adults and racial groups other than White, Black.

[a]Family structure and size estimates realigned to match population projections. See text for details. [b]Count of spouse, biological and stepchildren.

Table S6. Projected Number of U.S. Adults Ages 65 and Older with Care Needs by Number and Type of Caregivers Due to Population Growth Only and Changes in Age, Family Structure and Family Size: 2022, 2030 and 2040 (Millions)

	Main Projections				Alternative Projections			
	2022	2030	2040	% Increase	2022	2030	2040	% Increase
Number with No Caregivers Assuming:								
Population Growth Only	4.9	5.8	6.0	22.9%	5.4	6.3	6.6	22.9%
& Age Structure Changes	4.8	5.8	6.2	27.8%	5.3	6.4	6.8	27.8%
& Family Structure Changes	4.9	5.8	6.0	23.6%	5.4	6.5	6.7	24.2%
& Family Size Changes	4.9	5.8	6.1	23.7%	5.4	6.4	6.6	24.0%
Number with Only Paid Caregivers Assuming:								
Population Growth Only	0.7	0.8	0.8	22.9%	0.9	1.1	1.1	22.9%
& Age Structure Changes	0.7	0.8	0.9	40.5%	1.0	1.3	1.5	58.8%
& Family Structure Changes	0.7	0.8	0.8	25.7%	0.9	1.1	1.2	24.8%
& Family Size Changes	0.7	0.8	0.8	25.6%	0.9	1.1	1.2	27.3%
Number with One Family Caregiver Assuming:								
Population Growth Only	5.3	6.2	6.5	22.9%	8.0	9.5	9.9	22.9%
& Age Structure Changes	5.3	6.4	7.0	31.2%	8.2	9.8	10.8	32.8%
& Family Structure Changes	5.3	6.2	6.5	22.8%	8.1	9.7	10.0	23.3%
& Family Size Changes	5.3	6.3	6.5	23.4%	8.0	9.7	10.0	24.8%
Number with Two Family Caregivers Assuming:								
Population Growth Only	2.2	2.6	2.7	22.9%	3.5	4.1	4.3	22.9%
& Age Structure Changes	2.2	2.8	3.4	52.9%	3.6	4.4	5.2	45.5%
& Family Structure Changes	2.2	2.6	2.7	21.3%	3.5	4.1	4.3	23.9%
& Family Size Changes	2.2	2.6	2.7	22.0%	3.4	4.0	4.2	23.3%
Number with Three or More Family Caregivers Assuming:								
Population Growth Only	1.4	1.6	1.7	22.9%	2.8	3.3	3.4	22.9%
& Age Structure Changes	1.4	1.7	2.1	49.2%	2.9	3.7	4.4	52.0%
& Family Structure Changes	1.4	1.6	1.7	21.2%	2.8	3.2	3.3	19.6%
& Family Size Changes	1.4	1.6	1.7	22.2%	2.8	3.2	3.3	19.4%

Note: Main projections exclude foreign born older adults, Hispanic and racial groups other than White, Black. Alternative projections include these groups and assume they grow at the same rate as the baseline population.

Table S7. Distribution of Type and Number of Family Caregivers and Mean Number, Among those with Limitations, 2022 & 2040

	2022	2040: Age Structure changes	2040: Family Structure changes	2040: Family Size changes
None	26.1%	23.6%	26.2%	26.1%
Paid only	4.5%	5.4%	4.5%	4.7%
1 Family Caregiver	39.1%	37.6%	39.3%	39.4%
2 Family Caregivers	16.9%	18.1%	17.0%	16.6%
≥3 Family Caregivers	13.5%	15.2%	13.1%	13.1%
Mean number among those with limitations	1.1	1.2	1.1	1.1

Note: Projections exclude foreign born older adults, Hispanic and racial groups other than White, Black.

Table S8. Projected Number of U.S. Adults Ages 65 and Older with Care Needs and No Caregivers Due to Population Growth Only and Changes in Age, Family Structure and Family Size: 2022, 2030 and 2040 (Millions)

Number with No Caregivers Assuming:	2022	2030	2040	% increase
Population Growth Only	14.4	17.1	17.7	22.9%
& Age	14.3	17.1	18.0	25.9%
& Family Structure	14.3	16.9	17.6	23.3%
& Family Size	14.4	17.1	17.8	23.6%

Note: Projections use alternative definition of care need that includes all individuals with unassisted difficulty whether they report an unmet care need or not. Projections exclude foreign born older adults, Hispanic and racial groups other than White, Black.
